# Dr. Charles W. Nichols

**Published:** 1912-06

**Authors:** 


Dr. Charles W. Nichols of Whitesboro died April 23, 1912, of cerebral haemorrhage, aged 62. He was a graduate of Albany, 1889. He was a Civil War veteran and, for several years was health officer of Fairfield.

